# Single-Port Access Laparoscopy-Assisted Vaginal Hysterectomy: Our Initial Experiences with 100 Cases

**DOI:** 10.1155/2012/543627

**Published:** 2012-09-04

**Authors:** Young-Sam Choi, Kwang-Sik Shin, Jin Choi, Ji-No Park, Yun-Sang Oh, Tae-Eel Rhee

**Affiliations:** ^1^Department of Obstetrics and Gynecology, Eun Hospital, Duam-Dong, Buk-Gu, Kwang-Ju 500-101, Republic of Korea; ^2^Department of General Surgery, Eun Hospital, Duam-Dong, Buk-Gu, Kwang-Ju 500-101, Republic of Korea

## Abstract

*Objectives.* To present our initial experiences with laparoscopically assisted vaginal hysterectomy performed using homemade transumbilical single-port system. *Materials and Methods.* We reviewed the medical records of one hundred patients who underwent single-port access laparoscopically assisted vaginal hysterectomy (SPA-LAVH). SPA-LAVH was performed with homemade single port system and conventional rigid laparoscopic instruments. *Results.* All procedures were successfully completed through the single-port system and vagina without need for extraumbilical puncture or conversion to laparotomy. The median patient age was 48.2 ± 6.5 years. Thirty-three patients had history of past abdominopelvic surgery. The median total operative time, largest dimension of the uterus, and weight of the uterus were 73.1 ± 24.6 min, 10.5 ± 2.1 cm, and 300.8 ± 192.5 gram, respectively. The median decline in the hemoglobin from before surgery to postoperative day 1 was 1.8 ± 0.9 g/dL. Bladder injury in occurred one patient who was repaired through intraoperative laparoscopic suture. The postoperative course was uneventful in most patients except for three who had a transient paralytic ileus, five who had pelvic hematoma, but they were recovered following conservative managements. No port-related complications were noted, and the cosmetic results were excellent. *Conclusions.* SPA-LAVH is technically safe procedure, and the homemade single-port system offers reliable access for single-port surgery.

## 1. Introduction

To optimize the benefits of minimally invasive procedures, surgeons have attempted to reduce the overall abdominal wall trauma by decreasing either the size of the ports or the number of trocars.

In these efforts, transumbilical single-port surgery uses an umbilical single incision technique to access the peritoneal cavity and target organs.

Owing to the nature of umbilicus, single-port laparoscopy through the umbilicus offers an exciting opportunity to perform laparoscopic surgery with no visible scar.

However, transumbilical single-port laparoscopy is not a new concept in gynecologic surgery [1–5].

In 1969, Wheeless and Thompson first published the technique and the results of a large series of laparoscopic tubal ligations using single-trocar laparoscopy. Later, Wheeless reported a large series of one-incision tubal ligation.

Additionally, in 1991, the first laparoscopic total abdominal hysterectomy with bilateral salpingooophorectomy (BSO) using only a single incision was reported by Pelosi and Pelosi III. One year later, four supracervical hysterectomies with BSO for benign uterine disease were reported by the same authors [1–5].

Although single-port surgery enhances cosmetic benefits and reduces postoperative pain and morbidity, use of this technique was not widespread due to technical difficulties. However, with advances in instrumental and surgical skills, the technical difficulties associated with this surgical procedure have been overcome considerably [6–15].

Particularly, single-port surgery is ideal for laparoscopic-assisted vaginal hysterectomy (LAVH) because the vagina of woman can be considered as an additional route for surgery; thus, uterine manipulators can be applied through the vagina [11–17].

Unlike uterine repair following myomectomy or bowel reanastomosis after bowel resection, SPA-LAVH does not require a reconstruction process through a single port. This is because the vaginal stump can be repaired not by laparoscopy, but through the vagina.

In this study, we report our initial 100 cases observations of SPA-LAVH (with or without bilateral salpingooophrectomy (BSO)) using a homemade, single-port, three-channel system.

## 2. Materials and Methods

### 2.1. Data Analysis

A retrospective medical records review was performed for the initial 100 patients who underwent SPA-LAVH at Eun hospital.

Between March 2010 and September 2011, 100 patients had undergone SPA-LAVH for nonmalignant gynecological diseases, including uterine leiomyoma (25 cases), adenomyosis (19 cases), adenomyosis coexisting leiomyoma (41 cases), preinvasive lesion of cervix coexisting adenomyosis or leiomyoma (7 cases), ovarian huge cyst (5 cases), endometrial hyperplasia (2 cases), and tuboovarian abscess (1 case).

Past abdominopelvic surgery, body mass index (BMI), and the size of the uterus were not considered as exclusion criteria.

The following parameters were determined in the present observational study: age, parity, BMI, surgical history, indication for surgery, operative time (from incision to final umbilical closure), largest dimension of the uterus, weight of the extirpated uterus (as pathology report), hemoglobin change (from before surgery to postoperative day 1), and perioperative and postoperative complications.

### 2.2. Operation Procedures

We used homemade, single-port, three-channel system using the Alexis wound retractor (Applied Medical, Rancho Santa Margarita, CA, USA), surgical glove, two 10 mm trocars, and one 5 mm trocar [7, 16, 17].

After partial eversion of the umbilicus, a curved semilunar skin incision was performed at the hidden lateral aspect of the umbilical crater. The incision was C-shaped and followed the natural curve of the inferior lateral aspect of the umbilical crater near the base. After skin incision, a rectus fasciotomy and peritoneal incision were performed by direct cut-down technique.

An approximately 1.5 2 cm-sized skin incision was sufficient to install the three-channel, single-port system, because of the elasticity of the skin and the tissue beneath it, which can be dissected as long as required [16, 17].

**Figure 1:**
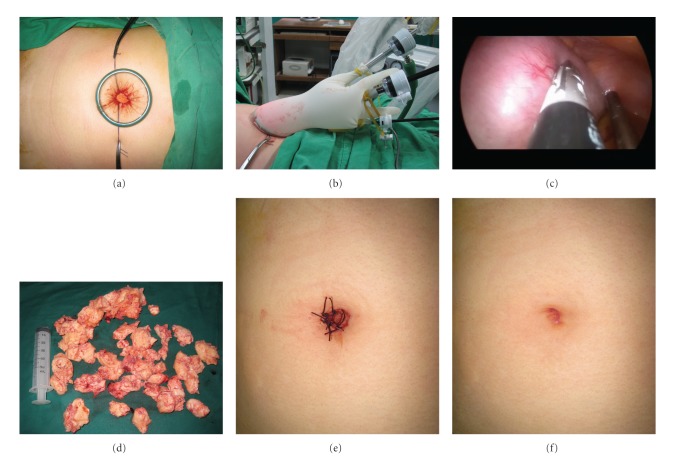
SPA-LAVH for adenomyosis with coexisting myoma (46-year-old woman). (a) Transumbilical single route for surgery using Alexis wound retractor. Distal ring was loaded within the intraperitoneal space and tightly turned inside out of the proximal ring, creating an effective seal and a wider opening of the single-port incision by connecting retractable sleeve between the distal and proximal rings. The fascial edges were tagged with suture for traction prior to port system installation; this was useful for fascial closure at the end of the procedure. (b) Homemade, three-channel, single-port system using the Alexis wound retractor and a surgical glove. A sterile surgical glove was placed over the proximal ring and fixed tightly, and three trocars were inserted through surgical glove with cut edges of the distal fingertips and tied with an elastic string. Varying the height of the trocar head minimized clashing of the bulkiest portion of the trocar head and the instrumental grip (the external handle) extracorporeally overlapping. (c) Laparoscopic finding: huge uterine leiomyoma with coexisting adenomyosis. The largest dimension of the uterus was 15 cm. (d) Photograph showing an extirpated uterus. The weight of the uterus was 750 g. Compared with 50 mL disposable syringe. (e,f) Photograph showing the postoperative umbilical skin wound (postoperative day 1 and 4 weeks).

As shown in Figure 1(a), the fascial edges were tagged with suture for traction prior to port system installation; this was useful for fascial closure at the end of the procedure.

The Alexis wound retractor consists of a proximal ring, distal ring, and connecting retractable sleeve.

As shown in Figure 1(a), the distal ring was loaded within the intraperitoneal space and tightly turned inside out of the proximal ring (rolled up manner), creating an effective seal and a wider opening of the single-port incision by connecting retractable sleeve between the distal and proximal rings.

Once fixed in the opening site, it laterally retracted the sides of the wound opening. This made the small incision as a wider and rounder opening.

Subsequently, as shown in Figure 1, a sterile surgical glove was placed over the proximal ring and fixed tightly to prevent leakage of carbon dioxide gas. Three trocars were inserted through the surgical glove with cut edges of the distal fingertips and tied with an elastic string. The elastic nature of the glove enabled to achieve an airtight seal, which maintained the pneumoperitoneum. The multiple truncated fingers of the glove functioned as a multiport for surgical instruments [16, 17].

The use of instruments with different overall positions was also helpful.

A limited range of motion was closely related to the bulkiest portion of the trocar head and instrumental grip (external handle) extracorporeally overlapping.

As shown in Figure 1(b), the length of the instruments was the same, but the lengths of the truncated glove digits varied. However, varying the height of the trocar head may minimize clashing of the bulkiest portion of the trocar head and instrumental grip (the external handle) extracorporeally overlapping.

All the surgical procedures were performed as a standard LAVH (with or without BSO) technique using conventional nonarticulated rigid laparoscopic instruments and the LigaSure system (Valleylab, Boulder, CO, USA).

As has been established earlier, exploration of pelvis, coagulation and cut of ligaments and vessel above the uterine vessel, and bladder mobilization were undertaken in laparoscopic phase.

Ligation of uterine vessel, cardinal and uterosacral ligament, extirpation of uterus, and vaginal stump closure were undertaken in the vaginal phase.

Subsequently, the laparoscope was used to check the pelvis for hemostasis.

## 3. Results

All procedures were successfully completed through the single-port system and vagina without the need for extraumbilical puncture or conversion to laparotomy. As shown in Table 1, the mean ± standard deviation (SD) of patient age, parity, and BMI was 48.2 ± 6.5 years, 2.3 ± 1.0, 25.4 ± 3.3 kg/m^2^, respectively. Thirty-three patients had a past history of abdominopelvic surgery, such as a Caesarean section, laparoscopic tubal ligation, appendectomy, ovarian cystectomy, or salpingooophrectomy. Among these patients, six had a history of Caesarean sections, five had a history of repeat Caesarean sections, and five had a history of three Caesarean sections. Seven patients needed 2-3 units of packed red blood cell transfusion due to chronic anemia or intraoperative hemorrhage. The mean ± SD of time to installation of the transumbilical single-port system was 7.3 ± 1.5 min. The mean ± SD of total operative time, largest dimension of the uterus, and weight of the uterus were 73.1 ± 24.6 min, 10.5 ± 2.1 cm, and 300.8 ± 192.5 gram, respectively.

The operative time between laparoscopic phase and vaginal phase was similar but depended on pelvic pathology.

The median decline in the hemoglobin level from before surgery to postoperative day 1 was 1.8 ± 0.9 g/dL. Bladder injury occurred in one patient who had a history of three Caesarean sections but was repaired through intraoperative laparoscopic suture.

The postoperative course was uneventful in most patients, but three had a transient paralytic ileus, and five had pelvic hematoma, all of whom recovered following conservative managements. No port-related complications were noted, and the cosmetic results and patient satisfaction were excellent.

## 4. Conclusion

SPA-LAVH is a technically safe and feasible procedure, and the homemade single-port system offers reliable and cost-effective access for single-port surgery.

## 5. Discussion

As mentioned earlier, LAVH is most ideal for single-port surgery because the vagina of woman can be considered as an additional route for surgery; thus, uterine manipulators can be applied through the vagina.

Unlike uterine repair after myomectomy, LAVH does not require a reconstruction process through a single port. This is because the vaginal stump can be repaired not by laparoscopy, but through the vagina.

Thus, SPA-LAVH is safe, and the procedure can be learned by skillful surgeons over a short period of time, because a considerable portion of the procedure can be performed through the vagina. The homemade three-channel, single-port system using a surgical glove and an Alexis wound retractor offers reliable, flexible, and cost-effective access for single-port procedures, and the system can be applicable in nonarticulated, rigid, conventional laparoscopic instruments [16, 17].

Limitations of single-port surgery include the loss of instrumental triangulation, reduced operative working space, reduced laparoscopic visualization, and instrumental crowding and clashing.

These limitations act as hurdles for some reconstructive procedures, such as repair after myomectomy.

However, the reconstructive procedure can be performed with instrumental advancement, such as the use of articulated instruments [6–15].

Our observations show that a history of abdominopelvic surgery is not a contraindication for single-port surgery; however, central obesity is problematic to secure a route for the single-port system through a small intraumbilical incision. Procedural difficulties resulting from previous abdominopelvic surgery are not because of the single-port surgery itself, but owing to abdominopelvic conditions [10, 15–17].

A linear correlation existed between the operation time and an extirpated uterine weight of >400 g, because more time was needed for uterine fragmentation for extirpation through the vagina; however, no linear correlation existed between the operation time and a uterus weight of <400 g. For pelvic adhesion, such as in previous pelvic surgery or endometriosis, additional operation time is required for adhesiolysis.

This study has several limitations. It is not a case-control study, and pain score, hospital stay, cost effectiveness, and return to work were not considered because of the retrospective nature of the study.

Additional clinical data and long-term followup may be needed to address port-related complications.

## Figures and Tables

**Table 1:** Clinical data and surgical outcomes of SPA-LAVH (*N* = 100).

	Demographic characteristics	Median ± SD*	Range*
Preoperative characteristics	Age (years)	48.2 ± 6.5	36–68
	Parity	2.3 ± 1.0	0–5
	Body Mass Index (kg/m^2^)	25.4 ± 3.3	18.8–36.5
	Past abdominopelvic surgery	Caesarean section	6
		Repeat Caesarean sections	5
		Three times Caesarean sections	5
		Tubal ligation	9
		Appendectomy	3
		Appendectomy and tubal ligation	2
		Ovarian cystectomy	2
		Unilateral salpingooophrectomy	1
	Indication for hysterectomy	Leiomyoma	25
		Adenomyosis	19
		Adenomyosis coexisting leiomyoma	41
		Preinvasive lesion of cervix coexisting adenomyosis	7
		Adnexal disease	5
		Endometrial hyperplasia	2
		Others	1

Intraoperative course	Time to installation of single-port system (min)	7.3 ± 1.5	5–13
	Total operative time (min)	73.1 ± 24.6	33–180
	Largest dimension of uterus (cm)	10.5 ± 2.1	6–15
	Weight of uterus (gram)	300.8 ± 192.5	90–1007
	Extraumbilical puncture	0	
	Conversion to laparotomy or conventional multiport laparoscopy	0	
	Great vessel injury	0	
	Bowel injury	0	
	Bladder injury	1	Intraoperative repair
	Ureter injury	0	
	Blood transfusion	7	

Postoperative course	Hemoglobin drop (g/dL)	1.8 ± 0.9	0.5–4.4
	Pelvic hematoma	5	Conservative management
	Sepsis	0	
	Return to operation room	0	
	Transient paralytic ileus	3	Conservative management
	Thromboembolic events	0	
	Cosmetic effects	Excellent	
	Port-related complications	0	

*Values are presented as mean ± standard deviation or absolute number.
